# A commentary on “Evaluation of the comparative efficacy and safety of surgical strategies for long bone defects: a network meta-analysis”

**DOI:** 10.1097/JS9.0000000000002739

**Published:** 2025-06-24

**Authors:** Mm Fei Jiang, Mm He Hu

**Affiliations:** Department of Orthopedics, Dalian Women and Children’s Medical Group, Liaoning, China

*Dear Editor,*

Bone defects that not heal naturally are widespread in medical practice and are frequently caused by osteomyelitis, trauma, or tumor resection procedures^[1]^. However, clinical surgeons have an even greater challenge when soft tissue injury or infections exacerbate these bone defects. The Papineau technique, Masquelet technique, Ilizarov method, vascularized bone grafting, autologous cancellous bone grafting, and allograft transplantation are some of the efficient methods now available for treating bone defects^[2,3]^. It is essential to determine if the fracture can mend properly and whether the patient can attain satisfactory functional results after surgery before choosing a treatment plan for bone defects. Strategies for managing potential postoperative complications, such nonunion, must also be taken into account.

A network meta-analysis was conducted by Shen *et al*^[4]^ to assess the effectiveness and safety of several surgical treatments for long bone defects. There were 20 observational studies and three randomized controlled trials comprising 930 individuals. The findings revealed that bone transport and vascularized bone graft could provide better clinical results for long bone defects. There is a significant risk of complications with bone tissue engineering.

Regardless of the recipient bed’s blood supply, vascularized bone graft can provide full blood supply by arteriovenous anastomosis after transplantation. By just needing the fusing of the graft’s ends, it avoids the requirement for new bone callus development and mineralization, which lowers the rate of bone resorption and speeds up healing and treatment time. Nevertheless, it also provides limited bone availability for significant deformities, necessitates precise and intricate surgical methods, and requires a high degree of microsurgical experience.

According to a review, when treating critical-sized tibial defects, such as those larger than 8 cm, the Ilizarov bone transfer approach exhibits high union rates and decreased complication rates. Although vascularized bone graft and bone transport offer some benefits, it is crucial to note that future research may alter the ranking of therapies based on absolute and cumulative impact probabilities in network meta-analysis. Large-scale randomized controlled studies are therefore still required to confirm these results.

Several limitations of this study should be considered: (1) since observational studies do not employ random allocation, there is an inherent risks of residual confounding and bias; (2) owing to limited head-to-head studies, some treatments are more dependent on indirect evidence, which may introduce uncertainty; (3) the lack of standardized long-term follow-up data and functional outcome measures restricts the ability to comprehensively evaluate the effect of treatment on patients’ quality of life; (4) there were huge heterogeneities regarding the patient characteristics, rehabilitation program, and pharmacological intervention, thus, subgroup analysis should be performed to reduce the heterogeneities; (5) a significant geographical bias may have been introduced because the majority of the selected research were from Asian countries. Future research in other countries and regions should confirm the conclusion.

This article complies with TITAN Guidelines 2025^[5]^.

## Data Availability

None.
